# A Step Toward Eradication of Human Filariases in Areas Where *Loa* Is Endemic

**DOI:** 10.1128/mBio.00456-16

**Published:** 2016-04-12

**Authors:** Timothy G. Geary

**Affiliations:** Institute of Parasitology, McGill University, Ste-Anne-de-Bellevue, QC, Canada

## Abstract

Mass drug administration (MDA) programs have achieved remarkable success in limiting the pathology and transmission of the human parasitic infections onchocerciasis and lymphatic filariasis. The full implementation of MDA campaigns for filariasis elimination has been stymied by the unacceptable incidence of severe adverse events observed following drug treatment of a subset of individuals who harbor high loads of *Loa loa* microfilaria. Extending MDA strategies to regions where loiasis is coendemic could be done confidently if a simple, inexpensive, and rapid diagnostic method was available that could accurately identify individuals who have *L. loa* microfilarial loads above the risk threshold and could thus be excluded from treatment. A recent paper in *mBio* reports the discovery of an antigen unique to *L. loa* microfilaria that can be detected in blood and urine and may form the basis for such an assay. Further work will reveal whether this discovery will smooth the path to achieve filariasis eradication.

## COMMENTARY

The remarkable achievements made by filariasis control programs testify to the success possible with focused effort and investment in tropical health interventions. Human infections with *Onchocerca volvulus*, the cause of onchocerciasis, or river blindness, and *Wuchereria bancrofti*, the major cause of lymphatic filariasis, have been the subjects of prolonged mass drug administration (MDA) and vector control campaigns that have led to marked decreases in the pathology, transmission, and incidence of these diseases (1, 2). Local eradication has been achieved in some areas for both, with the notable example of the all-but-complete elimination of onchocerciasis from the Americas (3).

MDA campaigns distribute drugs that eliminate the microfilarial (MF; larval) stages of these long-lived nematode parasites for many months after a single dose. Diethylcarbamazine (DEC) is used along with albendazole for the control of lymphatic filariasis in areas where other filarial infections are not coendemic. Ivermectin (IVM) is used for onchocerciasis and for lymphatic filariasis in regions where both infections occur. Removing microfilariae blocks the transmission of both parasites and reduces the pathology of onchocerciasis. These strategies have worked remarkably well, with one important exception: both drugs may cause severe adverse events (SAEs), including death, in individuals harboring heavy MF burdens of *Loa loa*, another filarial nematode (4–6). Because of this, onchocerciasis control programs have been limited in regions where loiasis occurs; clinical experience has shown that individuals with burdens of >30,000 MF/ml blood are at particular risk of IVM-related SAEs (4–8).

Infections with *L. loa* have generally been considered to cause little to no overt pathology (7, 8). The distribution of *L. loa* coincides with that of *O. volvulus* in important parts of West and Central Africa (6–8), a factor that looms as an impediment to the timely eradication of *O. volvulus* infection via expanded IVM-based MDA campaigns in these areas (9). As the considerable majority of people infected with *L. loa* have MF levels well below the risk threshold (5–8), control through chemotherapy could be ramped up if a rapid and convenient diagnostic platform capable of identifying at-risk patients and, hence, excluding them from treatment, could be devised and implemented. For safety reasons, the threshold for inclusion of patients in an IVM treatment regimen is usually set at 30,000 MF/ml (5–8).

It is against this background that the new work by Drame et al. (10) stands out as a highly significant advance. Diagnosis of MF loads in loiasis patients has typically been done by counting MF in a small sample of blood under a microscope, a method that is invasive, time consuming, and sometimes difficult to use for quantification. The Nutman laboratory at the United States NIAID has been at the forefront of developing innovations to improve the detection and quantification of *Loa* MF, including nucleic acid-based methods (11). For use in the field, a loop-mediated isothermal amplification (LAMP) method is most advantageous in this category, since thermocycling is not required (11). This technique can identify individuals with high MF loads (>30,000/ml) but requires training and is still invasive, as it requires a blood sample to obtain MF. An impressive advance in convenience, cost, and time was the development of an off-the-shelf hand-held cell counter (12). Blood passing through size gates in this instrument leads to sequestration of the MF in a format that allows quantification. The technique is very rapid but, again, is invasive. Recently, this group described a rapid and sensitive technique for *Loa* MF quantification based on a cell phone microscope with software that can determine MF abundance based on pixel changes in two sequential recordings (13). It also provides a rapid method to identify individuals who should be excluded from IVM treatment, but this method also requires a blood sample.

The major advance in the work reported by Drame et al. (10) is eliminating the need for invasive procedures by identifying a biomarker of *Loa* MF present in urine. Based on the insight that some proteins can escape the renal filtering process, the authors searched for *Loa* proteins in urine obtained from a loiasis patient. Proteins of >3 kDa were precipitated with acetone and subjected to tryptic digestion. The resulting peptides were separated by reverse-phase liquid chromatography (LC) and submitted for tandem mass spectrometry (MS) analysis. A number of candidate *Loa* proteins were identified by this approach, all of which were found in MF. Filtering through bioinformatics screens eliminated proteins with significant homology to human proteins or proteins from other filariae and allowed the authors to prioritize a single *Loa* MF protein, termed LOAG_16927, which is unique to *L. loa* and has no predicted function.

The authors then developed a novel enzyme-linked immunosorbent assay (ELISA) to quantify the levels of this antigen in plasma based on a modification of the luciferase immunoprecipitation system (LIPS) format (14); using defined amounts of recombinant LOAG_16927 for competition, the authors developed a competitive LIPS assay that provided a reasonable correlation between antigen abundance and MF density in samples obtained from loiasis patients. This is the first potentially quantitative, MF-specific, antigen-based diagnostic test described for filariases and, as such, represents a significant advance. As the authors note, however, a considerable amount of work remains to be done to bring this assay to ready-to-use status for a field setting. In particular, it will have to be shown to be advantageous in terms of sensitivity, cost, time, or convenience compared to other techniques for quantifying *L. loa* MF in patient blood samples.

In addition to these important data, this paper suggests another intriguing avenue for research. It would be highly interesting to pursue the possibility that MF loads in loiasis patients could be quantified by an antigen detection assay using a urine sample. It is encouraging to note that a point-of-care circulating cathodic antigen (POC-CCA) test is commercially available for the urine-based diagnosis of schistosomiasis (see reference 15), suggesting that global health applications of such tests are feasible. That assay is not intended to be accurately quantitative, whereas a test for the *L. loa* MF burden would need to distinguish individuals with MF loads in the high-risk category. For use in loiasis control programs, it is likely that a host protein with biochemical characteristics similar to those of LOAG_16927 would have to be included to control for hydration status and urine volume. Nonetheless, avoiding the need to take a blood sample could be an important step forward for the identification of individuals who should be excluded from IVM treatment in onchocerciasis campaigns. It is also possible that a urine-based assay would find value for population-based epidemiological analyses, an application that would not require rigorous quantification of the MF burden. While a significant proportion of *L. loa* infections seem to be amicrofilaremic (5, 8), the convenience of a noninvasive assay is an attractive feature for further investment in this area. An additional attraction of a urine-based assay is that the levels of MF appearing in the peripheral blood vary considerably over time, typically peaking in the early afternoon (16). Since MF are not thought to leave the bloodstream during periods of sequestration from the peripheral circulation, urinary antigen loads might be less variable than MF counts in blood as determined by microscopic or molecular methods. It would be well worth the investment needed to subject this possibility to experimental testing.

Additional applications of technology to detect filarial proteins in urine are readily apparent. As eradication programs proceed, it will be important to identify infected individuals in areas of low endemicity to allow targeted treatment when MDA campaigns are no longer justified. The current surveillance techniques for infected humans involve invasive procedures, including a blood sample for lymphatic filariasis and skin snips for onchocerciasis. Although MF stages of the causative parasites are not found in the blood, unlike loiasis, it is possible that a urinary protein unique to *W. bancrofti* or *O. volvulus* could be identified and an assay developed to generate yes/no responses in a urine sample. In fact, a protein that has homologs in all filariae of humans but not in other kinds of helminth parasites could also be useful, simply to identify affected individuals for more detailed parasitological analysis. Quantitative analyses would not be needed for such population surveys.

As Drame et al. point out, the development of a cheap, reliable platform capable of quantifying LOAG_16927 in a way that is diagnostically valid and economically valuable will be challenging. This work is nonetheless an exceptionally promising advance that merits additional urgent investment to complete its translation to practice. The sooner we have a method to allow the confident extension of filariasis control programs to areas where loiasis is coendemic with other filarial infections, the faster the feasible goal of eradication can be met.
